# Pyroelectric nanoplates for reduction of CO_2_ to methanol driven by temperature-variation

**DOI:** 10.1038/s41467-020-20517-1

**Published:** 2021-01-12

**Authors:** Lingbo Xiao, Xiaoli Xu, Yanmin Jia, Ge Hu, Jun Hu, Biao Yuan, Yi Yu, Guifu Zou

**Affiliations:** 1 College of Energy, Soochow Institute for Energy and Materials Innovations, and Key Laboratory of Advanced Carbon Materials and Wearable Energy Technologies of Jiangsu Province Soochow University, 215006 Suzhou, China; 2 School of Science, Xi’an University of Posts & Telecommunications, 710121 Xi’an, China; 3 School of Physical Science and Technology & Jiangsu Key Laboratory of Thin Films, Soochow University, 215006 Suzhou, China; 4 School of Physical Science and Technology, ShanghaiTech University, 201210 Shanghai, China

**Keywords:** Heterogeneous catalysis, Carbon capture and storage, Ferroelectrics and multiferroics

## Abstract

Carbon dioxide (CO_2_) is a problematic greenhouse gas, although its conversion to alternative fuels represents a promising approach to limit its long-term effects. Here, pyroelectric nanostructured materials are shown to utilize temperature-variations and to reduce CO_2_ for methanol. Layered perovskite bismuth tungstate nanoplates harvest heat energy from temperature-variation, driving pyroelectric catalytic CO_2_ reduction for methanol at temperatures between 15 °C and 70 °C. The methanol yield can be as high as 55.0 μmol⋅g^−1^ after experiencing 20 cycles of temperature-variation. This efficient, cost-effective, and environmental-friendly pyroelectric catalytic CO_2_ reduction route provides an avenue towards utilizing natural diurnal temperature-variation for future methanol economy.

## Abstract (web summary)

CO_2_ is a problematic greenhouse gas, although its conversion to alternative fuels represents a promising approach to limit its long-term effects. Here, the authors demonstrate that CO_2_ can be reduced to methanol through pyroelectric catalysis under temperature variation near room temperature.

## Introduction

For hundreds of years, fossil fuels have been the main energy source for human activities and industrial manufacture. With the development of human society, the decrease in fossil energy and the increase of CO_2_ concentration have aroused great attention. For instance, energy crisis, greenhouse effect and ocean acidification are some of the main problems facing humanity^1–3^. Converting CO_2_ into hydrocarbon fuels is considered as one of the ideal solutions, which can solve not only the environmental problems but also the high requirements of energy consumption. Various methods have been explored to convert CO_2_ to organic fuels, such as photocatalytic reduction, electrocatalytic reduction, biological transformation, hydrogenation, and dry reforming^4–7^. Nevertheless, hydrogenation of CO_2_ to form CH_3_OH process requires high operating temperatures (200–250 °C) and high pressures (5–10 MPa), which limit the yield of methanol^8^. As a matter of fact, photocatalytic reduction of CO_2_ can be carried out at mild temperature and pressure, but it does not work in dark^9^.

Temperature variation is a recurring phenomenon in our daily life^10^. It would be meaningful to harvest such abundant energy source during temperature variation. Such a motive is reasonable because pyroelectric materials can convert heat energy into electric energy via repeating cooling or heating process^11–13^. Pyroelectric materials can produce positive and negative electric charges during temperature variation. The free charges generated through pyroelectric process can be applied to catalytic processes such as dye decomposition^14–16^ and water splitting^17,18^. Theoretical calculation shows that a pyroelectric engine in an ideal condition can reach an energy conversion efficiency as high as 84–92%, which is much higher than the photovoltaic energy conversion efficiency typically in the range of 20%^19,20^. Theoretically, Kakekhani et al.^21^ have proved the feasibility of pyroelectric catalytic water splitting. However, to our best knowledge, there is no report about collecting the energy using pyroelectric materials from temperature variation for CO_2_ reduction.

The catalytic performance of ferroelectrics has been studied for 70 years. For example, the internal fields from the polarization of the ferroelectrics can separate electrons and holes, thus enhancing the catalytic efficiency^17^. Ferroelectric polarization can affect molecular adsorption and desorption from the surface of the materials^21^. It is well known that all ferroelectric materials are pyroelectric materials. As the simplest member of bismuth layer-structured Aurivillius phase, bismuth tungstate (Bi_2_WO_6_) exhibits good ferroelectric and pyroelectric properties. Meanwhile, Bi_2_WO_6_ has some other interesting properties such as high ion conductivity, large spontaneous polarization (*P* ≅ 50 µC cm^−2^), high Curie temperature (*T*_C_ = 950 °C), and photocatalytic property^22,23^. As Bi_2_WO_6_ is constructed by alternating (Bi_2_O_2_)^2+^ and (WO_4_)^2−^ layers, such a layered structure enables high thermal and chemical stabilities^24^. More importantly, the suitable energy band structure and surface properties of Bi_2_WO_6_ allow it for CO_2_ reduction into renewable hydrocarbon fuel^25,26^. In this work, through pyroelectric catalysis, CO_2_ is reduced to CH_3_OH at temperature variation below 100 °C. The efficiency has reached as high as 55.0 µmol g^−1^ after experiencing 20 cycles between 15 °C and 70 °C. Our experimental work provides a new route to CO_2_ reduction for methanol through a pyroelectric catalytic process, which can be carried out near room temperature.

## Results and discussion

### Characterization of Bi_2_WO_6_

**Fig. 1:**
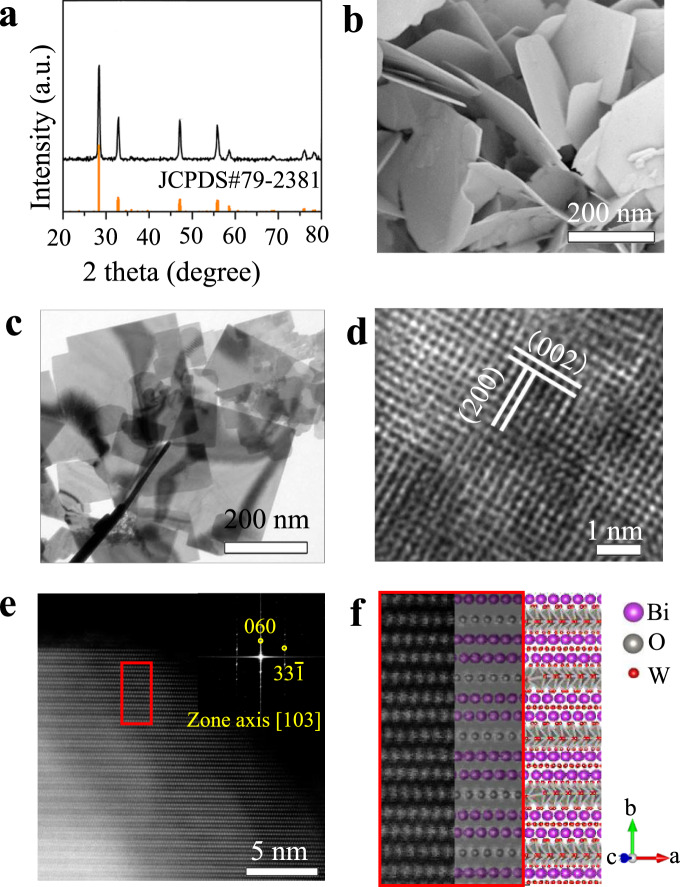
Structures of Bi_2_WO_6_ sample. **a** XRD pattern, **b** SEM photograph, **c** TEM photograph, **d** HRTEM image, and **e** Aberration-Corrected HAADF-STEM image. The inset image in **e** denotes the FFT of STEM. **f** Comparison between the STEM image and the structure model of the layered structure of Bi_2_WO_6_.

Previous study shows that Bi_2_WO_6_ is ferroelectric with an orthorhombic structure^27^. Bi_2_WO_6_ nanoplates were synthesized by hydrothermal process in this work (see the experimental details in the section of Methods). In order to identify the phase of the synthesized Bi_2_WO_6_, X-ray diffraction (XRD) analysis was performed at room temperature. As shown in Fig. 1a, all the diffraction peaks can be assigned to Bi_2_WO_6_ according to the standard JCPDS card No. 79-2381 (space group: *Pca2*_*1*_; point group: *mm2*; orthorhombic crystal system).

It can be seen from Fig. 1b that the synthesized Bi_2_WO_6_ has a plate-like morphology with an average size of 250 nm. Figure 1c presents the image of Bi_2_WO_6_ from transmission electron microscopy (TEM), where the nanoplate has the similar feature size as the ones of scanning electron microscope (SEM) images in Fig. 1b. The high-resolution transmission electron microscopy (HRTEM) image of Bi_2_WO_6_ is shown in Fig. 1d. It clearly shows the single-crystalline nature of Bi_2_WO_6_ nanoplate with a lattice plane intervals of 0.27 nm, corresponding to the (002)/(200) plane, respectively. The aberration-corrected high-angle annular dark field scanning transmission electron microscopy (HAADF-STEM) image of Bi_2_WO_6_ sample is presented in Fig. 1e. The light/dark gray contrast spots correspond to Bi (*Z* = 83, where *Z* is the atomic number) and W (*Z* = 74) atom columns, respectively. The inset image denotes the fast Fourier transformation (FFT) of the STEM image. The FFT image shows the zone axis of the STEM image is [103], which is perpendicular to *b* direction. Therefore, the STEM image reflects the layered structure of Bi_2_WO_6_, which is sandwiched by alternating perovskite-like (WO_4_)^2−^ and fluorite-like (Bi_2_O_2_)^2+^ blocks. A comparison between the STEM image and the structure model is schematically illustrated in Fig. 1f. The left picture in Fig. 1f is the magnified image of the area marked in a red rectangle in Fig. 1e. The inset in Fig. 1e shows the simulated diffraction pattern in the [103] projection direction. Complete structure model is shown on the right side of Fig. 1f.

**Fig. 2:**
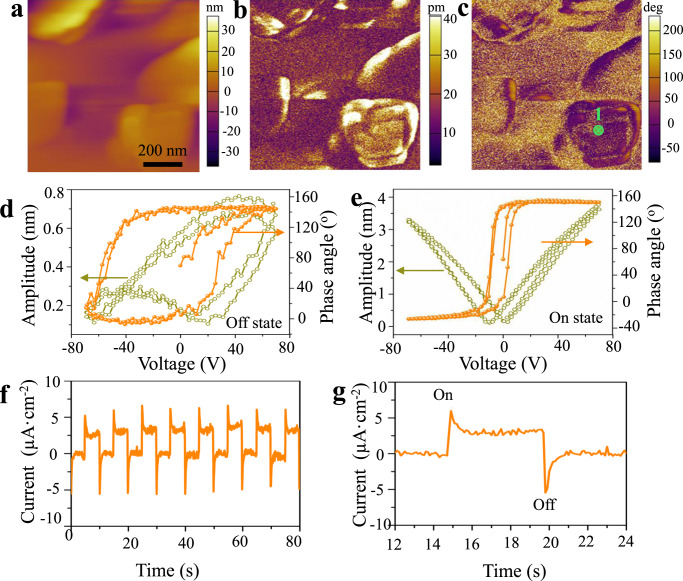
Ferro-/pyroelectric properties of Bi_2_WO_6_. **a** Topology, **b** vertical amplitude, and **c** phase images of Bi_2_WO_6_ nanoplate. The local hysteresis loops of Bi_2_WO_6_ nanoplate for point 1 marked in **c**: **d** “off” state, and **e** “on” state. **f** The Pyro-current response of Bi_2_WO_6_, **g** enlarged view of one full light on/off cycle is shown in **f**.

Bi_2_WO_6_ is paraelectric with a high-symmetry body-centered tetragonal structure (space group symmetry *I4/mmm*) at high temperature. When the temperature drops, symmetry of the crystal structure will be broken, and the distortion of the symmetry tetragonal structure makes Bi_2_WO_6_ generate ferroelectric properties. This mainly includes two aspects. First, the ions displace along the [110] axis of the tetragonal structure. Secondly, the WO_6_ octahedra rotates around the *a* and *c* axes^28^. In order to characterize the ferroelectric properties of the as-synthesized Bi_2_WO_6_ nanoplates, ferroelectric domains of Bi_2_WO_6_ nanoplates are observed using a piezoelectric force microscope (PFM) at a slow scanning frequency of 1 Hz with an area of 0.8 × 0.8 μm^2^. The nanoplates’ morphologies of Bi_2_WO_6_ in Fig. 2a are consistent with the results of TEM and SEM. Figure 2b, c show the vertical piezoresponse amplitude and phase image, respectively. The distinct contrast in the images illustrates the different polarization in the Bi_2_WO_6_ nanoplates. Figure 2d, e display the local piezoelectric hysteresis loops of the Bi_2_WO_6_, including both “off” state (piezoelectric displacement contribution only) and “on” state (both the piezoelectric contribution and the displacement resulted from electrostatic interaction). The phase angles at “off” and “on” states change about 150° under 60 V DC bias field, confirming the occurrence of a local polarization switching under an electric field. The butterfly-shaped hysteresis loop further verifies the local ferro-/piezoelectric response of Bi_2_WO_6_ nanoplate.

It is noted that Bi_2_WO_6_ also shows pyroelectric properties, where imbalanced polarization charges can generate electric field when the material undergoes temperature variation. The voltage produced by pyroelectric effect can be driving force for electrochemical reactions. Figure S1 shows the pyro-potential distribution across a Bi_2_WO_6_ nanoplate fitted by COMSOL finite element simulation, in which different colors represent different potentials. It can be seen that potential difference occurs on the surfaces of the Bi_2_WO_6_ nanoplates. In general, ferroelectric materials have greater pyroelectric and piezoelectric coefficients than non-ferroelectrics^28^. In order to demonstrate Bi_2_WO_6_ generate free charges through temperature variation, pyro-current response of Bi_2_WO_6_ nanoplates is measured. Once the temperature of the pyroelectric material changes, the pyroelectric charges can be generated quickly due to the pyroelectric effect. The pyroelectric current can be expressed by Eq. (1),

1 $$I_{{\mathrm{pyro}}} = p \cdot A \cdot \left( {{\mathrm{d}}T/{\mathrm{d}}t} \right)$$

where *I* and *p* are the pyroelectric current and the pyroelectric coefficient of Bi_2_WO_6_, respectively. *A* is the area of electrode. d*T*/d*t* is the rate of temperature fluctuation. Therefore, the pyroelectric current is proportional to d*T*/d*t*, any temperature change of the pyroelectric material will cause it to generate free charges. Figure 2f, g show the current change generated by Bi_2_WO_6_ nanoplates with the infrared signal. Under infrared radiation, a sharp increase of current density is induced by the pyroelectric effect due to the rapid increase of temperature within Bi_2_WO_6_ nanoplates. The current density decays slowly due to the decrease of temperature change rate, and maintains at a steady value under the equilibrium condition. When the infrared radiation is turned off, due to the instantaneous temperature decrease (d*T*/d*t* < 0), the redistribution of the pyroelectric charges will produce a reverse current. The output current returns to zero while there is no temperature change and infrared radiation. To clarify the temperature effect, we further use xenon lamp (UV light) instead of the infrared radiation to illuminate the sample. There is no pyro-current signal generate, which further confirms that Bi_2_WO_6_ generates free charge under temperature variation(see Fig. S2).

### Pyroelectric catalytic activity of Bi_2_WO_6_

**Fig. 3:**
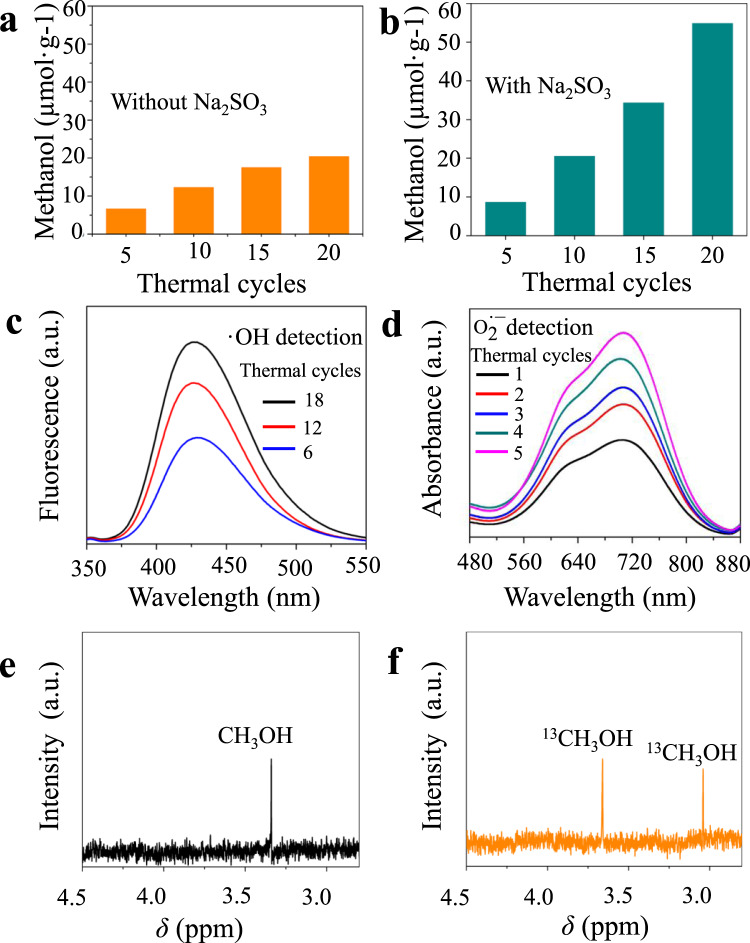
Catalytic activities of Bi_2_WO_6_. **a** Methanol yield through pyroelectric catalytic CO_2_ conversion without Na_2_SO_3_, and **b** with Na_2_SO_3_ as sacrificial agent. **c** The fluorescence spectra of 2-hydroxyterephthalic acid, **d** the absorption spectra of diformazan and monoformazan. **e** 1H NMR spectra of the pyroelectric catalytic reaction solution with unlabeled CO_2_ and **f** 1H NMR spectra of the pyroelectric catalytic reaction solution with labeled ^13^CO_2_.

In order to evaluate the pyroelectric catalytic activity of Bi_2_WO_6_, CO_2_ reduction test was carried out under the condition of temperature variation. As shown in Fig. 3a, the methanol yield increases with the increasing thermal cycles. The total methanol yield reaches 20.5 µmol g^−1^ without adding any sacrificial agent after 20 thermal cycles. ^1^H NMR in Fig. S3 also demonstrates that no other products can be detected in the liquid phase. Meanwhile, the analyses of gaseous products in Fig. S4 show only a small amount of CH_4_ and CO (0.11 µmol g^−1^ and 0.20 µmol g^−1^, respectively), indicating high selectivity of Bi_2_WO_6_ pyroelectric catalytic CO_2_ reduction to CH_3_OH. The oxygen production detection is shown in Fig. S5, the amount of O_2_ is roughly 1.5 times the amount of methanol. It has been similarly reported that the photocatalytic process works due to the recombination of electrons and holes. Such a process will significantly affect the catalytic efficiency^29,30^. To reduce the occurrence of electrons recombination with holes, sacrificial agents are usually added to the reaction system. Figure 3b shows that more methanol can be generated by using Na_2_SO_3_ as negative charge sacrificial agent. The methanol yield can be as high as 55.0 µmol g^−1^ after 20 temperature-variation cycles, which is 2.5 times more than that without Na_2_SO_3_. The range of temperature variation can also affect the pyroelectric catalytic CO_2_ reduction. Figure S6 shows the yield of methanol after 10 thermal cycles in different temperature ranges (15–40 °C, 15–50 °C, 15–70 °C, 15–85 °C). In Fig. S6, the methanol yield increases as the temperature range increases. The pyro-induced charges (d*Q*) can be expressed in Eq. (2)

2 $${\mathrm{d}}Q = {{p}} \cdot {{A}} \cdot {\mathrm{d}}T$$

A larger temperature range can generate more pyro-charges, leading to better pyroelectric catalytic results. It is also noted that the Bi_2_WO_6_ nanoplates maintain their crystal structure and morphology after pyroelectric catalytic reduction as confirmed by the XRD analysis and SEM characterization (see Fig. S7). To further prove that CO_2_ reduction comes from pyroelectric catalysis, experiment without Bi_2_WO_6_ nanoplates is performed, the result in Fig. S8a shows that no methanol or other products can be detected under temperature variation without Bi_2_WO_6_ nanoplates. Furthermore, no methanol or other products can be detected when the test is carried out with the presence of Bi_2_WO_6_ nanoplates for 10 h at temperatures of 15 °C, 45 °C and 70 °C, respectively (Fig. S8b). CO_2_ is a linear molecule, which is one of the most thermodynamically stable carbon compounds, it’s hard to break the bonding of C=O^8,31^.

Pyroelectric charges can react with O_2_ and OH^−^ in water to form ${\mathrm{O}}_2^{ \cdot - }$ and $\cdot {\mathrm{OH}}$. Such reactions can be expressed as shown in Eqs. (3)–(5).

3 $${\mathrm{Bi}}_2{\mathrm{WO}}_6\mathop { \to }\limits^{{{\Delta }}T} {\mathrm{Bi}}_2{\mathrm{WO}}_6{\mathrm{(}}q^ + + q^ - {\mathrm{)}}$$

4 $${\mathrm{O}}_2 + q^ - \to {\mathrm{O}}_2^{ \cdot - }$$

5 $${\mathrm{OH}}^ - + q^ + \to \cdot {\mathrm{OH}}$$

To have a better understanding of the pyroelectric catalysis, ${\mathrm{O}}_2^{ \cdot - }$ and $\cdot {\mathrm{OH}}$ detections are performed. Experimentally, the $\cdot {\mathrm{OH}}$ can be detected by fluorescence spectrometry using terephthalic acid as a photoluminescent $\cdot {\mathrm{OH}}$ trapping agent. The ${\mathrm{O}}_2^{ \cdot - }$ can be detected by UV–Vis spectrophotometer since ${\mathrm{O}}_2^{ \cdot - }$ can react with nitro-blue tetrazolium (BNT) to produce diformazan and monoformazan. As shown in Fig. 3c, significant fluorescence emission at ~425 nm associated with 2-hydroxyterephthalic acid is observed upon the temperature-variation cycles. The gradual increase of luminescence intensity with temperature-variation cycles indicates the formation of $\cdot {\mathrm{OH}}$. Figure 3d presents the absorption spectra of diformazan and monoformazan, which are produced by BNT reacted with ${\mathrm{O}}_2^{ \cdot - }$. The increase of peak absorption at ~630 nm and 720 nm with the temperature-variation cycles indicates the formation of ${\mathrm{O}}_2^{ \cdot - }$^32^. The electron spin resonance characterization in Fig. S9 can further prove ${\mathrm{O}}_2^{ \cdot - }$ and $\cdot {\mathrm{OH}}$ generated through temperature variation. ${\mathrm{O}}_2^{ \cdot - }$ and $\cdot {\mathrm{OH}}$ are considered to be the main active species in dye decomposition^33^. Except for the pyroelectric catalysis of CO_2_ reduction, we further performed RhB pyroelectric catalytic decomposition experiment to fully demonstrate the pyroelectric catalytic activities of Bi_2_WO_6_. In fact, RhB pyroelectric catalytic decomposition is a visual evidence to prove the redox ability of pyroelectric charges. Accordingly, Rhodamine B (RhB) solution (5 mg L^−1^) is used to demonstrate the pyroelectric catalytic dye decomposition of Bi_2_WO_6_ in Fig. S10a, b. In order to prove that methanol is the product of CO_2_ reduction, we manage the isotopic labeling experiment using ^13^CO_2_ as feedstock. To avoid ^12^CO_2_, we have done the additional experimental using NaOH instead of NaHCO_3_. The ^1^H NMR spectrum of the reaction solution (Fig. 3e) clearly shows the formation of methanol (*δ* = 3.34 ppm) when the unlabeled CO_2_ is used as feedstock. While using ^13^CO_2_ instead of CO_2_, the ^1^H NMR spectrum of the reaction solution in Fig. 3f shows doublet peaks between 3.7 and 3.0 ppm, which is attributed to the proton coupled with the ^13^C of ^13^CH_3_OH^34^. The results directly indicate that CO_2_ is the carbon source for the pyroelectric catalytic CO_2_ reduction into CH_3_OH. The time course change of the intensity is shown in Fig. S11.

### Theoretical calculation of CO_2_ reduction reaction path

**Fig. 4:**
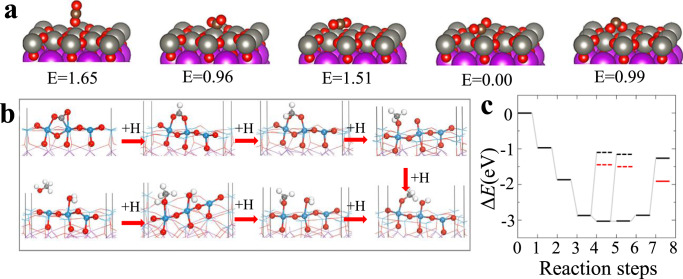
Adsorption configurations and reaction path of CO_2_ into CH_3_OH on Bi_2_WO_6_. **a** Five different adsorption configurations for CO_2_ on Bi_2_WO_6_. The red, gray, purple, and brown spheres stands for O, W, Bi, and C atoms. **b** Structures and **c** reaction energies for the CO_2_ reduction. Eight reaction steps are considered. The red, cyan, light gray, and dark gray spheres stand for O, W, H, and C atoms. Only the atoms around CO_2_ are highlighted. The reaction energies for the side product (methanol) at steps “4” and “5” are indicated by short dashed lines. The short red (dashed) lines are estimated from the free energies.

To better illustrate the reaction mechanism for the CO_2_ reduction, we employ first-principles calculations with SIESTA package, which is based on density functional theory (DFT)^35^. The pseudopotentials are constructed by the Troullier-Martins scheme^36^.The Ceperley–Alder exchange-correlation functional as parameterized by Perdew and Zunger is employed for the local density approximation (LDA)^37,38^. In all calculations, the double-ζ plus polarization basis sets are chosen for all atoms. The atomic structures are fully relaxed using the conjugated gradient method until the Hellman–Feynman force on each atom is smaller than 0.02 eV Å^−1^. Since Bi_2_WO_6_ consists of alternative (WO_4_)^2−^ and (Bi_2_O_2_)^2+^ layers as discussed above, a slab model is constructed for the Bi_2_WO_6_(001) surface. The top of the slab is terminated by the WO square network, and the bottom of the slab is saturated by H atoms, as shown in Fig. S12. It is known that oxygen vacancies commonly exist in oxide semiconductors^39^. However, it is found that the formation energy of an oxygen vacancy in Bi_2_WO_6_(001) is as large as 3.2 eV, which indicates that the density of oxygen vacancies in Bi_2_WO_6_(001) is ignorable. To explore the possible process of the CO_2_ reduction, it is necessary to figure out the ground-state adsorption configuration of the CO_2_ molecule on Bi_2_WO_6_(001). Figure 4a shows five different adsorption configurations for CO_2_. The lowest adsorption energy is −3.6 eV, which implies that the CO_2_ molecule is strongly bound to Bi_2_WO_6_(001). In this case, the CO_2_ molecule is bent with one C–W bond (2.00 Å) and two O–W bonds (2.09 and 2.26 Å) as shown in Fig. 4b (step “0”), which is different from previous report^9^. The C–O bond lengths are elongated by 0.1 Å because of the interaction between CO_2_ and Bi_2_WO_6_ (001). The CO_2_ reduction starts when the hydrogen ions in the solvent interact with the CO_2_ molecule. Note that DFT calculations, the hydrogen atom as proton (H^+^) and electron (e^−^) cannot be separated directly. To model the reaction between H and radical on Bi_2_WO_6_(001), a H atom is placed beside a certain site of the radical and carried out DFT calculations to optimize the interaction between them. Electron charge transfer happens between the H atom and radical, usually from H to the radical so that the H atom finally becomes H^+^, according to the chemical bonding between H and the radical (e.g., CO_2_ molecule in this work). In other words, charge separation can be reached after self-consistent-field iterations. In addition, the gas phase of H is assumed because the solvent does not involve in the reaction of the CO_2_ reduction. The process of the CO_2_ reduction is divided into a series of steps and the reaction energies are calculated step by step. All possible structural configurations along with addition of one H ion are considered for each step simulation. For instance, from step “0” to step “1”, the H ion may bind to the CO_2_ molecule through C or O atom, or to the Bi_2_WO_6_ (001) surface through W or O atoms, which results in different products. To determine the most possible reaction, the reaction energy of each product is estimated as: ${{\Delta }}E = E\left( n \right) - E\left( {n - 1} \right) - \mu _{\mathrm{H}}$, where $E\left( n \right)$ is the total energy of a certain product at the *n*^th^ step and $\mu _{\mathrm{H}}$ is the chemical potential of H. After optimizing all structural configurations, the case with the lowest reaction energy at each step is plotted in Fig. 4b. Obviously, the structural configurations in Fig. 4b are the most possible products for each step. Further, Fig. 4c shows the reaction energy of the most possible product at each step. Here, the first three H ions at the first three steps prefer to bind to the C atom, and these reactions are exothermic due to the large negative reaction energies as seen in Fig. 4c. As a consequence, one C–O bond is broken, then a CH_3_O^*^ radical and a separate O ion are produced at step “3”. Then the subsequent H ions will be attracted by the separated O ion until a H_2_O molecule forms (step “5”). However, the H_2_O molecule is not released from Bi_2_WO_6_ (001), because it requires a large activation energy of about 1.7 eV. Finally, a methanol (CH_3_OH) molecule is produced after one more H ion attaches to the CH_3_O^*^ radical (step “6”). As shown in Fig. 4c, the first four reaction steps are exothermic while the last three are endothermic. In particular, an activation energy of 1.6 eV (equal to the reaction energy) is needed for the CH_3_OH molecule to be detached from the Bi_2_WO_6_(001) surface, i.e., from step “6” to step “7”. Note that CH_3_OH may also be produced at steps “4” and “5” (short dashed lines in Fig. 4c), but the corresponding activation energies are 1.77 and 1.87 eV, respectively, even larger than that for step “7”. Therefore, the possibility to produce CH_3_OH at steps “4” and “5” is very small, because the major reactions at the first two steps are exothermic. Nevertheless, the overall process of the CO_2_ reduction is still exothermic as shown in Fig. 4c, that is, the activation energies in the last three reaction steps can be compensated by the energy released in the first four steps. In principle, the CO_2_ reduction happens spontaneously. However, energy supply might be required while the energy loss in a solvent environment. This is the reason that the temperature is not high in our experiments. It is worth pointing out that, when the CH_3_OH molecule is detached from the Bi_2_WO_6_ (001) surface, the zero-point energy and enthalpy contribute to the free energy significantly^40^. Therefore, the reaction energies are also estimated through the free energies for CH_3_OH production indicated by the red lines in Fig. 4c. Interestingly, the activation energy of step “7” decreases to 0.95 eV, which implies that the reaction may happen at relatively high temperature, as found in our experiments.

**Fig. 5:**
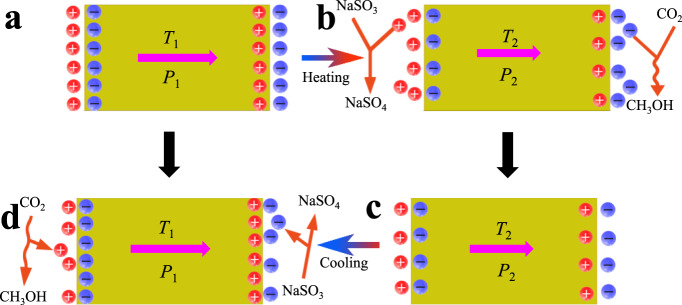
The mechanism of pyro-catalytic CO_2_ reduction induced by pyroelectric Bi_2_WO_6_ nanoplate. **a** The temperature of catalyst remains constant, its spontaneous polarization in equilibrium with the bound charges. **b** The rapid rise in temperature broke the balance, and thus induce CO_2_ reduction reaction. **c** A new balance is established after the CO_2_ reduction reaction. **d** When the temperature drops, the opposite charges transfer, leading to a new CO_2_ reduction process.

On the basis of the above analysis, the mechanism of pyroelectric catalytic CO_2_ reduction induced by temperature variation is proposed as shown in Fig. 5. When the temperature of Bi_2_WO_6_ remains stable, the internal spontaneous polarization is balanced with the external bound charges (Fig. 5a). It has been reported that the spontaneous polarization intensity of pyroelectric materials decreases as its temperature increases^41^. That is to say, the increase in temperature will reduce the polarization of the pyroelectric catalyst, thereby breaking the balance and generating free charges. The free negative charges react with adsorbed CO_2_ to form methanol and the free positive charges would be captured by Na_2_SO_3_ to form Na_2_SO_4_ (Fig. 5b). As a result, balance is established again between polarization and bonding charges (Fig. 5c). On the other hand, the decrease of temperature causes the increase of spontaneous polarization, and the equilibrium will be broken again, thus leading to opposite charges transfer and CO_2_ reduction process (Fig. 5d). Then the catalyst temperature returns to its original value and at the same time it returns to its original equilibrium. Therefore, the continuous thermal cycles will cause continuous CO_2_ reduction reaction.

In summary, this work introduces the use of pyroelectric materials to reduce CO_2_ to methanol driven by temperature variation. Experimental results show that the yield of methanol generation from CO_2_ can be as high as 55.0 µmol g^−1^ after 20 cycles of temperature variation. This efficient and environmentally friendly process based on the pyroelectric nanomaterial Bi_2_WO_6_ provides great potential for CO_2_ reduction in utilizing environmental heat energy near room temperature.

## Methods

### Materials

All used chemicals are analytic grade reagents without further purification. Bismuth nitrate (Bi(NO_3_)_3_·5H_2_O, AR), sodium tungstate (Na_2_WO_4_·2H_2_O, AR) and sodium bicarbonate (NaHCO_3_, AR), dimethyl sulfoxide (DMSO, AR) were acquired from Sinopharm Chemical Reagent Co., Ltd. Sodium sulfite (Na_2_SO_3_, AR was purchased from Shanghai Macklin Biochemical Co., Ltd. Carban dioxide (CO_2_, ≥99.995%) was purchased from Soochow Jinhong Co., Ltd. Deuterium oxide (D_2_O, ≥99.9%) was purchased from Qingdao Asfirst Science and trade Co., Ltd. ^13^CO_2_ was bought from Soochow changyou gas Co., Ltd with purity of 99.9%. Deionized water was employed throughout the whole experiments.

### Preparation of Bi_2_WO_6_ nanoplates

Bi_2_WO_6_ nanoplates were synthesized through hydrothermal process. In a typical process, 485 mg of Bi(NO_3_)_3_·5H_2_O (1 mmol) and 165 mg of Na_2_WO_4_·2H_2_O (0.5 mmol) were added into the mixed solution. White precipitate appeared immediately in the solution. After being washed for several times, the collected precipitate was added into a 50 mL Teflon-lined autoclave and filled with deionized water up to 80% of the total volume. Then the autoclave was sealed into a stainless steel tank and kept at 433 K for 20 h. After reactions, the white as-prepared sample was centrifugated at 2400 × *g* and washed three times with deionized water. Finally, the collected products were dried in vacuum at 333 K for 12 h for further use.

### Characterization

The crystal structure was test by an X-ray diffractomer (Philips PW3040/60, the Netherlands) with monochromatic Cu K*α* radiation (*λ* = 1.5406 Å, 2*θ* = 20°−80°). The morphologies of the Bi_2_WO_6_ sample was characterized by a transmission electron microscope (TEM, Hitachi H-7650, Japan) and a field emission transmission electron microscopy (FETEM, Scios, USA) with an accelerated voltage of 200 kV. The high-resolution transmission electron microscopy (HRTEM) image was acquired through a field emission transmission electron microscopy (FEI Tecnai G2 F20 S-TWIN, USA) with the accelerated voltage of 200 kV. Aberration-corrected high-angle annular dark field scanning transmission electron microscopy image was obtained on a 300 kV aberration-corrected JEM-ARM300F. The piezoelectric property of the Bi_2_WO_6_ sample was characterized with piezoresponse force microscopy (PFM, MFP-3D, USA). Photoluminescence (PL) measurements were carried out with a Horiba spectrofluorometer (Fluoromax-4, France) in air. Pyro-current response was measured on a CHI 660E electrochemical workstation using a three-electrode cell. The UV–visible absorption spectra are recorded on a UV2501PC (Shimadzu, Japan).

### Pyroelectric catalytic CO_2_ reduction activity test

In the pyroelectric catalytic CO_2_ conversion process, Bi_2_WO_6_ powder (40 mg) was suspended in 5 mL 0.2 M NaHCO_3_ solution in a 50 mL flask with the addition of 0.3 M Na_2_SO_3_ as sacrificial donor. High purity CO_2_ gas was bubbled into the flask for 10 min. Then the flask was immediately sealed with a rubber stopper. Then the flask was immediately sealed with a rubber stopper. The sample was suspended in the solution under magnetic stirring, being applied alternating temperature between 15 °C and 70 °C in water bath. The entire catalytic process is performed in dark. The detailed temperature profile and schematic diagram of the process can be found from Figs. S13 and  S14, respectively. To detect the formation of methanol, 1 mL solution was fetched out and analyzed by using a gas chromatograph (Persee G5) equipped with a KB-5 column connected to a flame ionization detector. For the nuclear magnetic resonance (NMR) test, 800 μL reaction solution, 100 μL D_2_O and 50 μL DMSO (0.1% vol aqueous solution) were taken into nuclear magnetic tubes, and detected with an NMR spectrometer with superconducting magnet (AVANCE NEO 400 MHz, Switzerland).

## Supplementary information

Supplementary Information

Peer Review File

## Data Availability

The data that support the findings of this study are available from the corresponding author upon reasonable request.

## Source data

Source Data
