# Author Correction: Pyroelectric nanoplates for reduction of CO_2_ to methanol driven by temperature-variation

**DOI:** 10.1038/s41467-021-23801-w

**Published:** 2021-06-02

**Authors:** Lingbo Xiao, Xiaoli Xu, Yanmin Jia, Ge Hu, Jun Hu, Biao Yuan, Yi Yu, Guifu Zou

**Affiliations:** 1 College of Energy, Soochow Institute for Energy and Materials Innovations, and Key Laboratory of Advanced Carbon Materials and Wearable Energy Technologies of Jiangsu Province Soochow University, 215006 Suzhou, China; 2 School of Science, Xi’an University of Posts & Telecommunications, 710121 Xi’an, China; 3 School of Physical Science and Technology & Jiangsu Key Laboratory of Thin Films, Soochow University, 215006 Suzhou, China; 4 School of Physical Science and Technology, ShanghaiTech University, 201210 Shanghai, China

**Keywords:** Heterogeneous catalysis, Carbon capture and storage, Ferroelectrics and multiferroics

Correction to: *Nature Communications* 10.1038/s41467-020-20517-1, published online 12 January 2021.

The original version of this Article contained errors in Fig. 3a and Fig. 3b, in which the phrases “With Na_2_SO_3_” incorrectly appeared in Fig 3a and “Without Na_2_SO_3_” appeared in Fig 3b rather than the correct phrases “Without Na_2_SO_3_” in Fig 3a and “With Na_2_SO_3_” in Fig 3b. This has been corrected in both the PDF and HTML versions of the Article.

