# Supplementary material for: Pyroelectric nanoplates for reduction of CO2 to methanol driven by temperature-variation
Source: Nat Commun. 2021 Jan 12;12:318. doi: 10.1038/s41467-020-20517-1 (PMC7804252; doi:10.1038/s41467-020-20517-1)
Supplement: Supplementary file 1 — Supplementary Information [file 41467_2020_20517_MOESM1_ESM.pdf]

Supplementary Information

Pyroelectric catalytic CO_2 reduction for methanol driven by temperature-variation

Lingbo Xiao[1,†], Xiaoli Xu[1,†], Yanmin Jia[2,*], Ge Hu[3], Jun Hu[3,*], Biao Yuan,[4] Yi Yu,[4] Guifu Zou[1,*]

[1]*College of Energy, Soochow Institute for Energy and Materials Innovations, and Key Laboratory of Advanced Carbon Materials and Wearable Energy Technologies of Jiangsu Province Soochow University, Suzhou 215006, China;*

[2]*School of Science, Xi'an University of Posts & Telecommunications, Xi'an, 710121, China;*

[3]*School of Physical Science and Technology & Jiangsu Key Laboratory of Thin Films, Soochow University, Suzhou 215006;*

[4]*School of Physical Science and Technology, ShanghaiTech University, Shanghai, 201210, China.*

*E-mail: jiayanmin@xupt.edu.cn (Y. Jia), jhu@suda.edu.cn (J. Hu) and zouguifu@suda.edu.cn (G. Zou).

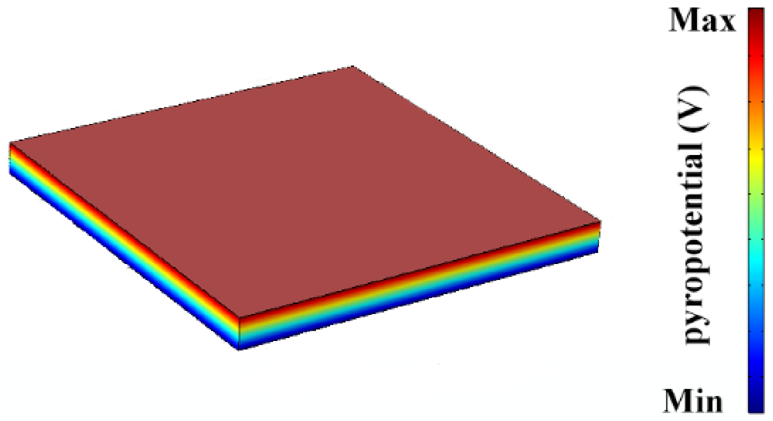

Fig. S1 The COMSOL simulation of pyro-potential of Bi_2WO_6 nanoplate, different colors represent different potentials. It can be seen potential difference occurs on the surfaces of the Bi_2WO_6 nanoplate.

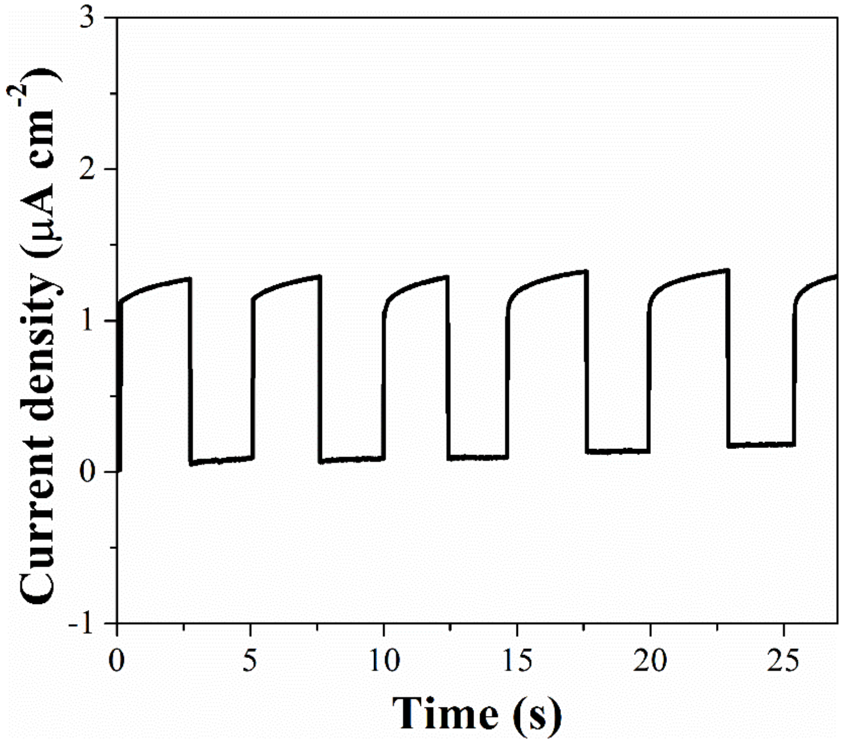

Fig. S2 Photoelectric response of Bi_2WO_6 excited by xenon lamp.

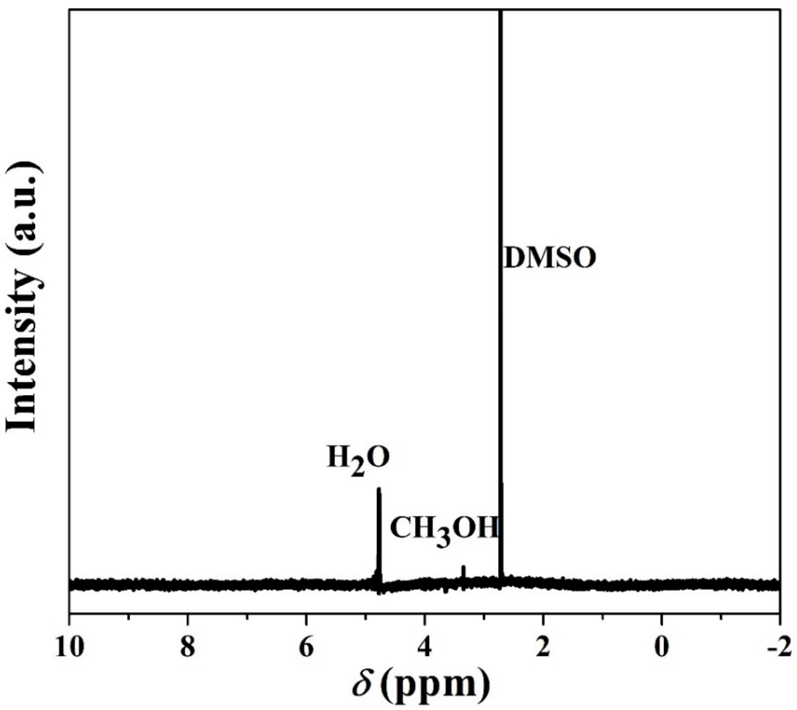

Fig. S3 ^{1}H NMR spectra of the pyroelectric catalytic reaction solution, the DMSO were added as internal standard reference.

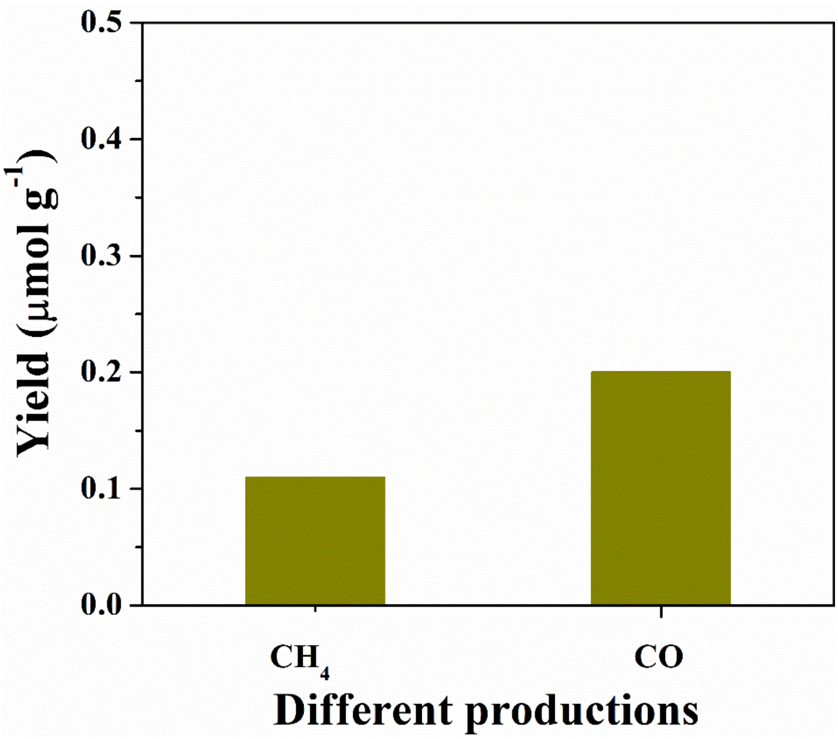

Fig. S4 Gas products by pyroelectric catalytic after 20 thermal cycles.

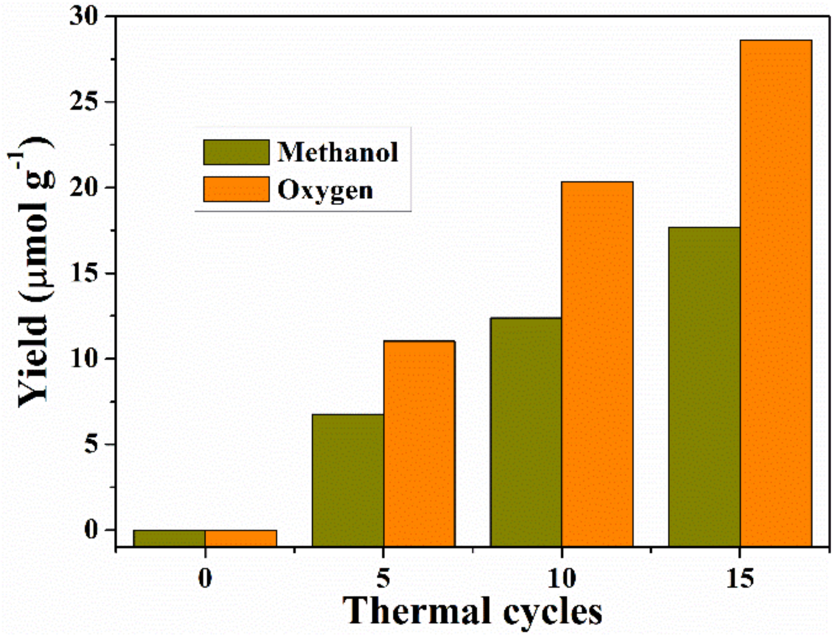

Fig. S5 Yield of Methanol and Oxygen without Na_2SO_3.

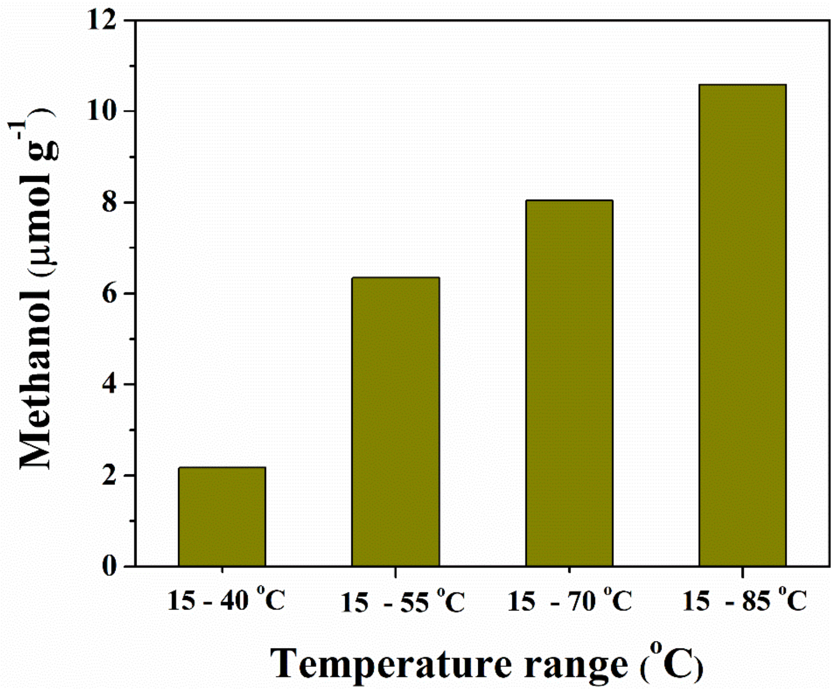

Fig. S6 Methanol yield of different temperature change ranges.

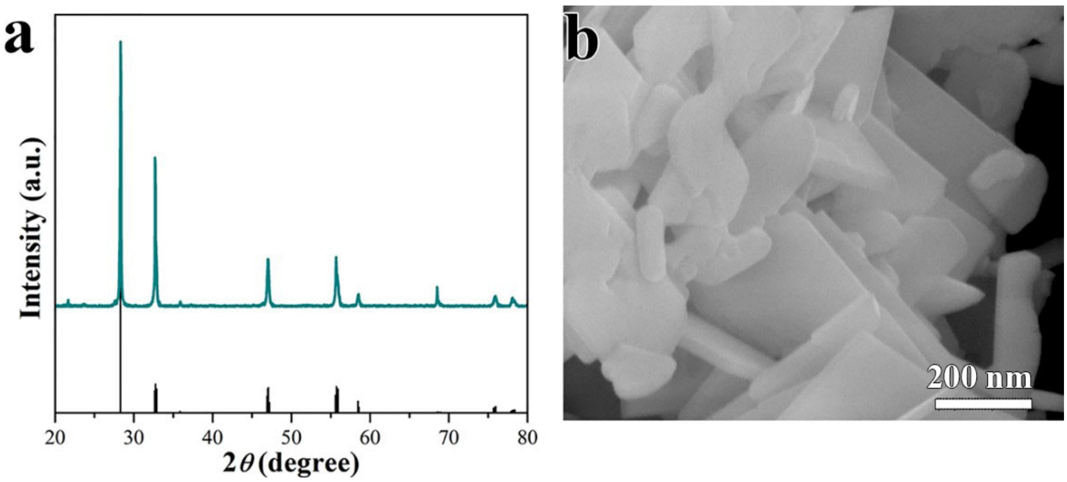

Fig. S7 Characterization of Bi_2WO_6 after pyroelectric catalysis. (a) XRD, (b) SEM.

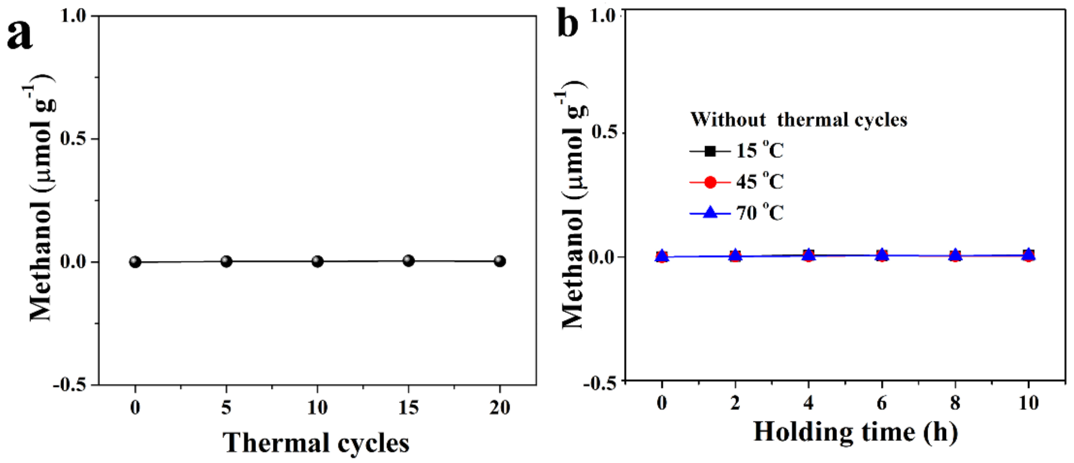

Fig. S8 Pyroelectric catalytic CO_2 reduction (a) without Bi_2WO_6 (b) with Bi_2WO_6 at different stable temperature.

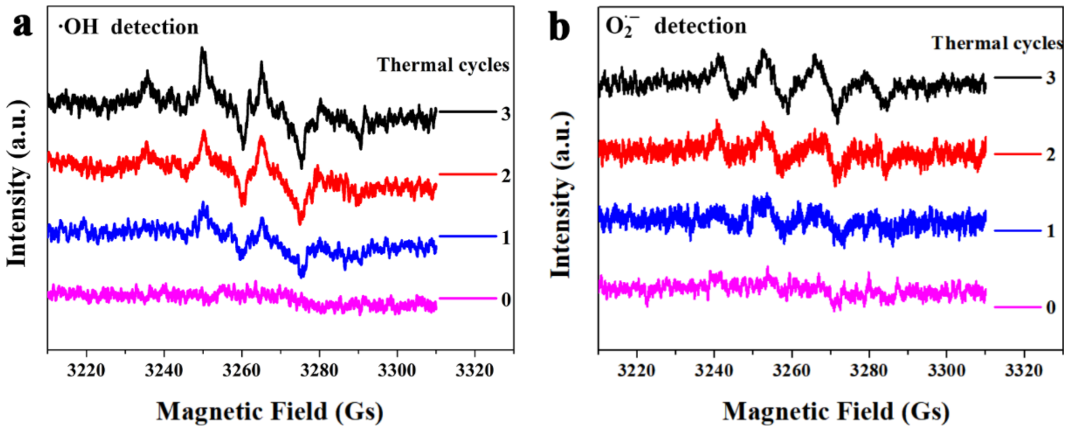

Fig. S9 Electron spin resonance spectra of (a) DMPO-·OH and (b) DMPO-$O_2^{\cdot-}$ adducts after undergoing different thermal cycles.

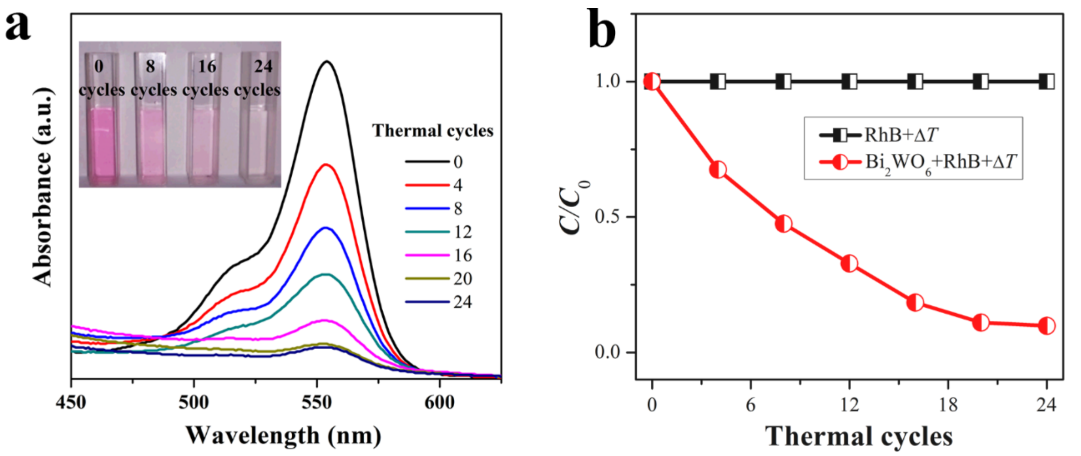

Fig. S10 (a) The absorption spectra of RhB in aqueous dispersion systems after experiencing different thermal cycles. The inset picture in (a) shows an intuitive diagram of RhB dye decomposition. (b) The pyroelectric catalytic decomposition efficiency of RhB with or without Bi_2WO_6 under different thermal cycles.

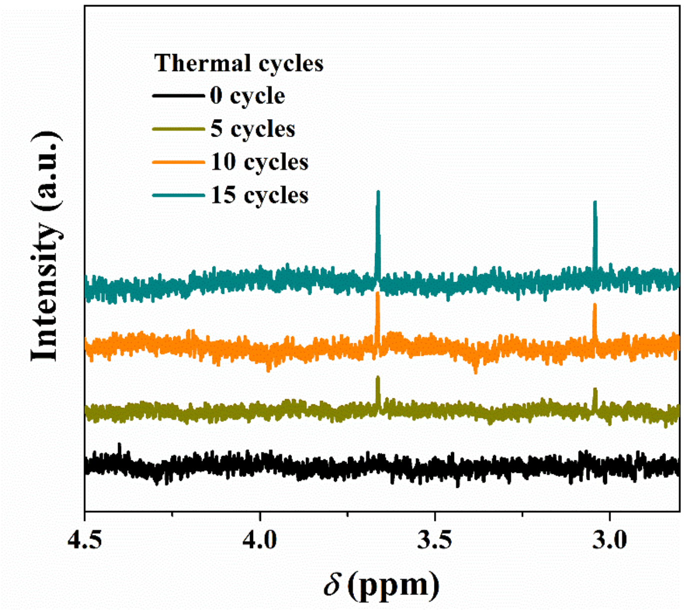

Fig. S11 NMR of isotope-label test with cumulative thermal cycling over time.

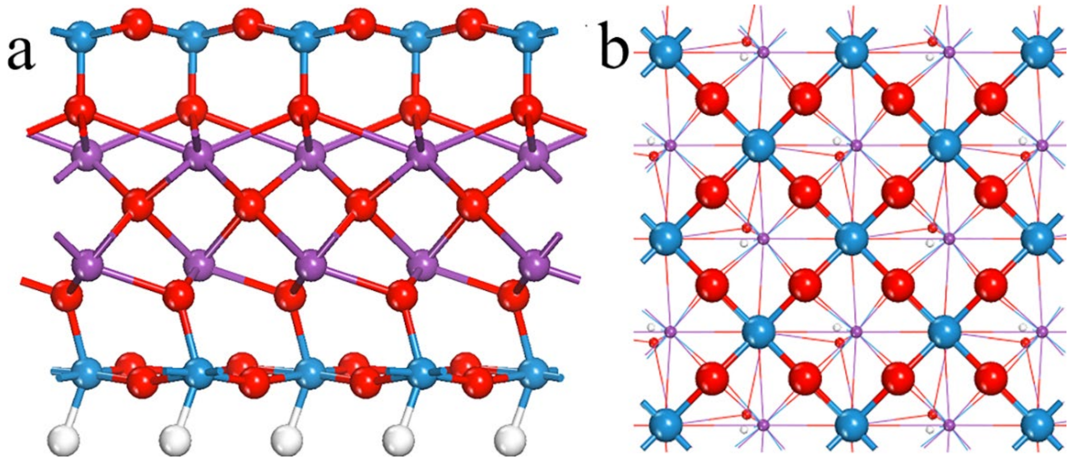

Fig. S12 Slab model for the Bi_2WO_6(001) surface. (a) and (b) Side and top views of the Bi_2WO_6(001) surface. The red, cyan, purple and light grey spheres stand for O, W, Bi and H atoms, respectively. In (b), the surface O and W atoms are highlighted to distinguish them from the atoms underneath.

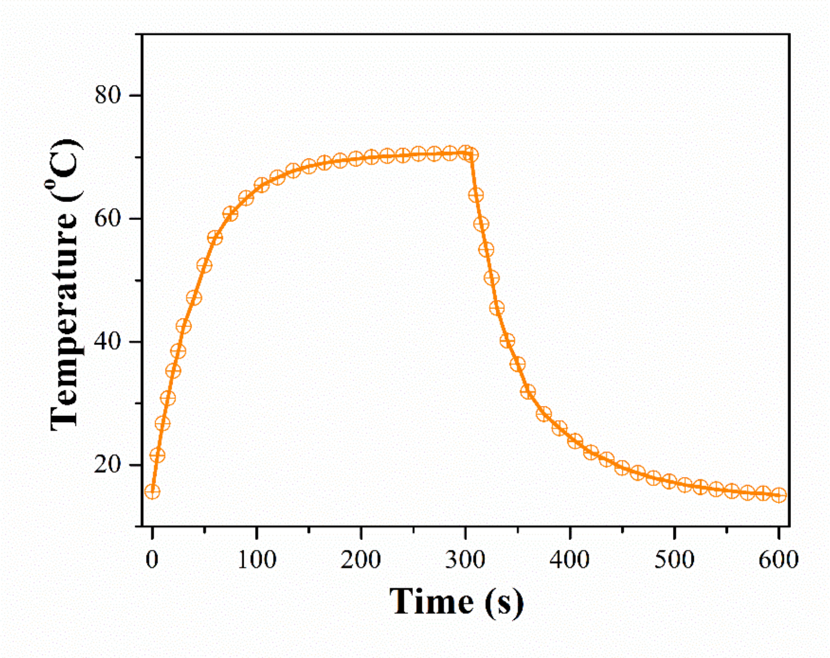

Fig. S13 Temperature variation between 15 -70 °C during the pyroelectric catalytic CO_2 reduction.

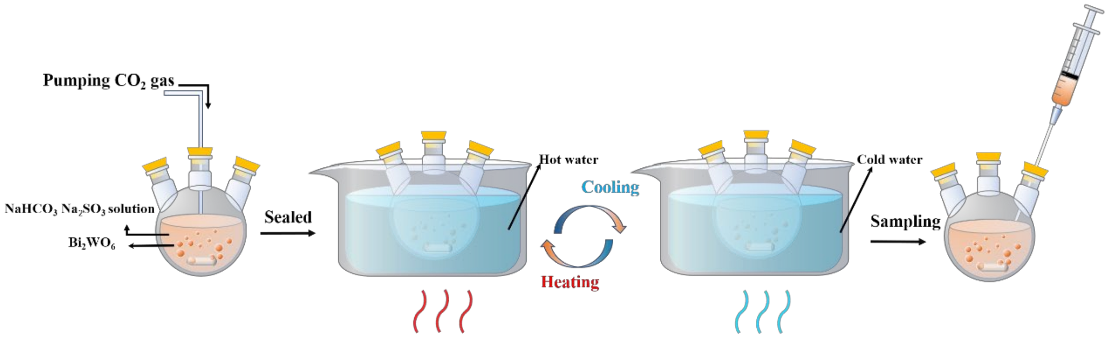

Fig. S14 Schematic diagram of catalytic reaction. The reaction solution is 0.2M $NaHCO_3$ and 0.3M Na_2SO_3 mixed aqueous solution. $NaHCO_3$ can be decomposed into CO_2 during heating, increasing the CO_2 content in the system, Na_2SO_3 is added as sacrificial donor. Then 40 mg Bi_2WO_6 powder was added as catalyst. After High purity CO_2 gas bubbled into the solution the flask was immediately sealed with a rubber stopper. The sample was suspended in the solution under magnetic stirring, then the flask is applied alternating temperature between 15 ºC and 70 ºC in water bath. The entire catalytic process is performed in dark. 1 mL solution was fetched out by injection syringe for further detection analysis.
